# The identification of a novel shared therapeutic target and drug across all insulin-sensitive tissues under insulin resistance

**DOI:** 10.3389/fnut.2024.1381779

**Published:** 2024-03-26

**Authors:** Jinyuan Xu, Lilin Zhu, Jie Xu, Kailong Lin, Juan Wang, Yan-long Bi, Guo-Tong Xu, Haibin Tian, Furong Gao, Caixia Jin, Lixia Lu

**Affiliations:** ^1^Department of Ophthalmology, Shanghai Tongji Hospital Affiliated to Tongji University, School of Medicine, Tongji Eye Institute, Shanghai, China; ^2^Department of Biochemistry and Molecular Biology, School of Medicine, Tongji University, Shanghai, China; ^3^Department of Genetics, Tongji University School of Medicine, Shanghai, China; ^4^Department of Ophthalmology of Ten People Hospital Affiliated to Tongji University, School of Medicine, Shanghai, China

**Keywords:** bioinformatics, insulin resistance, ubiquitin D, promethazine, insulin-sensitive tissues

## Abstract

**Background:**

To identify key and shared insulin resistance (IR) molecular signatures across all insulin-sensitive tissues (ISTs), and their potential targeted drugs.

**Methods:**

Three datasets from Gene Expression Omnibus (GEO) were acquired, in which the ISTs (fat, muscle, and liver) were from the same individual with obese mice. Integrated bioinformatics analysis was performed to obtain the differentially expressed genes (DEGs). Weighted gene co-expression network analysis (WGCNA) was carried out to determine the “most significant trait-related genes” (MSTRGs). Enrichment analysis and PPI network were performed to find common features and novel hub genes in ISTs. The shared genes of DEGs and genes between DEGs and MSTRGs across four ISTs were identified as key IR therapeutic target. The Attie Lab diabetes database and obese rats were used to verify candidate genes. A medical drug-gene interaction network was conducted by using the Comparative Toxicogenomics Database (CTD) to find potential targeted drugs. The candidate drug was validated in Hepa1-6 cells.

**Results:**

Lipid metabolic process, mitochondrion, and oxidoreductase activity as common features were enriched from ISTs under an obese context. Thirteen shared genes (Ubd, Lbp, Hp, Arntl, Cfd, Npas2, Thrsp., Tpx2, Pkp1, Sftpd, Mthfd2, Tnfaip2, and Vnn3) of DEGs across ISTs were obtained and confirmed. Among them, Ubd was the only shared gene between DEGs and MSTRGs across four ISTs. The expression of Ubd was significantly upregulated across four ISTs in obese rats, especially in the liver. The IR Hepa1-6 cell models treated with dexamethasone (Dex), palmitic acid (PA), and 2-deoxy-D-ribose (dRib) had elevated expression of Ubd. Knockdown of Ubd increased the level of p-Akt. A lowing Ubd expression drug, promethazine (PMZ) from CTD analysis rescued the decreased p-Akt level in IR Hepa1-6 cells.

**Conclusion:**

This study revealed Ubd, a novel and shared IR molecular signature across four ISTs, as an effective biomarker and provided new insight into the mechanisms of IR. PMZ was a candidate drug for IR which increased p-Akt level and thus improved IR by targeting Ubd and downregulation of Ubd expression. Both Ubd and PMZ merit further clinical translational investigation to improve IR.

## 1 Introduction

The global prevalence of obesity has reached pandemic levels (1). Insulin resistance (IR) is a common core pathophysiological basis for obesity, type 2 diabetes, and other metabolic diseases (2). Therefore, the identification of novel IR targets and target-interacting drugs can contribute to better prevention and treatment of IR. The insulin-sensitive tissues (ISTs) include brown adipose tissue (BAT), white adipose tissue (WAT), muscle, and liver. Enhancing the sensitivity of these ISTs to insulin is critical to improving systemic IR. Current studies suggest that inflammation, endoplasmic reticulum stress, and mitochondrial dysfunction are involved in the development of IR, however, the mechanisms underlying IR have not been fully understood (3–6).

Bioinformatics has become an integral part of biomedical science research and development, which involves the study, development, or application of computational tools and methods to acquire, store, visualize, and interpret medical or biological data (7). The increasing quantity of available datasets in public repositories such as Gene Expression Omnibus (GEO) and ArrayExpress, provides excellent opportunities to conduct comprehensive analyses by integrating multiple studies to identify robust molecular signatures that might be otherwise unidentifiable in individual studies (8). Currently, several relevant bioinformatics analyses of IR molecular signatures only focused on liver (9, 10) and WAT (11). Several genes, such as MYC, ANXA2, GDF15, AGTR1, NAMPT, LEPR, IGFBP-2, IL1RN, MMP7, and APLNR (9) and JUN, SERPINE1, GINS2, TYMS, HMMR, IGFBP2, BIRC3, TNFRSF12A (10) have been identified as IR signature in non-alcoholic fatty liver disease. While several genes, IL6, MMP9, CXCL8, CCL4, CXCL10, PTGS2, CCL2, SELE, CCL2, and BCL2A1 have been identified in WAT (11). However, there is no relevant study on the identification of shared IR signatures across four ISTs.

IR is a heterogeneous disease with a defect in the insulin signaling pathway. The insulin signaling pathway can be divided arbitrarily into proximal and distal segments. The distal segment refers to the substrates of AKT that are intimately linked to the various physiological functions of insulin and are often specific to a particular cell type. However, the proximal segment consists of the canonical elements, which include the insulin receptor, insulin receptor substrate proteins, phosphoinositide 3- kinase, and AKT. A unifying feature of the proximal components is that they contain considerable spareness such that a relatively small proportion of each element is required to evoke a physiological signal. Thus, the p-Akt level was considered to be the marker for insulin sensitivity. The identification of IR molecular signatures across ISTs is important for developing potential therapeutic targets for IR (3).

Unlike previous studies, the present study aimed to integrate IR-related datasets from four ISTs and perform appropriate meta-analyses to identify the shared IR molecular signatures. By bioinformatics analysis, *in vivo* and *in vitro* experiments, 13 shared molecular signatures (Ubd, Lbp, Hp, Arntl, Cfd, Npas2, Thrsp., Tpx2, Pkp1, Sftpd, Mthfd2, Tnfaip2, and Vnn3) of IR were identified and validated across four ISTs. Ubd was confirmed to be the strongest correlation between gene abundance and IR trait. Promethazine (PMZ), a candidate drug targeting Ubd, improved the decrease of p-Akt level in Hepa1-6 induced by Dex, PA, and dRib treatment. Both Ubd and the drug PMZ merit further clinical translational investigation to improve IR.

## 2 Methods

### 2.1 Data collection

Gene expression data related to IR were downloaded from the Gene Expression Omnibus (GEO) from the National Center of Biotechnology Information. The inclusion–exclusion criteria are described as follows: (i) all samples included these four tissues (BAT, WAT, Muscle, and Liver) simultaneously (control variables to reduce the occurrence of false positive results); (ii)datasets should include normal chow diet (NCD) and high-fat diet (HFD); and (iii) the datasets should contain three or more samples for each group.

### 2.2 Meta-analysis

The meta-analysis was performed according to our previous work (12). Briefly, after batch-effect adjustment, bioinformatics tools such as Networkanalyst (13) were used to screen the differentially expressed genes (DEGs), the KEGG Orthology-Based Annotation System platform (14) was used to perform enrichment analysis, the Search Tool for the Retrieval of Interacting Genes database (15) was used to construct the protein–protein interaction (PPI) networks, based on this, hub genes were identified using the CytoHubba plugin (16) in the Cytoscape software (version 3.7.2) (17).

### 2.3 Weighted gene co-expression network analysis (WGCNA)

To find modules highly correlated with IR, WGCNA was performed using the online analysis website imageGP^1^ and carried out on all genes (18). The strongest positive and negative correlation module genes were designated “most significant trait-related genes” (MSTRGs). The variance of gene expression in GSE15822 was calculated and the top 5,000 genes with large variation according to their variance for the WGCNA analysis were selected. The WGCNA was employed to test the independence and average connectivity of different modules under different β power values, and the β power values corresponding to an independence index of *R*^2^ = 0.85 were selected. The minimum size for module detection was 25. Potential correlations between modules and IR were explored through Pearson correlation analysis.

### 2.4 Drug-gene interaction network analysis

The Comparative Toxicogenomics Database (CTD)9, an online database providing information on the interactions between genes and drugs, and their relationships to diseases, was used to construct the medical drug-common genes interaction network (19).

### 2.5 Correlation between 13 shared genes and obese diabetes

Correlation between 13 common genes and obese diabetes was performed with the Attie Lab Diabetes database.^2^ The Attie Lab Diabetes database is a searchable resource of the gene expression data that is used to display the gene expression profiles of different experimental groups (lean and obese B6 mice at 4 and 10 weeks of age) in adipose tissue, muscle, and liver (20).

### 2.6 Experimental animals and obese model construction

Male Sprague–Dawley rats weighing 180 to 200 g were purchased from Slaccas (Shanghai, China). The obese rat model was prepared with 12 weeks of HFD feeding. Four ISTs were dissected and collected for subsequent experiments. The plasma was collected from NCD and HFD rats, respectively for subsequent untargeted metabolomics analyses (Lian Chuan Sciences, China).

### 2.7 Hematoxylin and eosin staining

Four ISTs were fixed with 4% paraformaldehyde for 24 h. After dehydration and transparent treatment, they were soaked in wax for embedding. The wax pieces were sliced (3 μm) and HE stained according to our previous work (12).

### 2.8 Cell lines and IR cell model

The mouse liver cell line Hepa1-6 was purchased from the American Type Culture Collection. The hepa1-6 was maintained in Dulbecco’s modified Eagle’s medium/high glucose, supplemented with 10% fetal bovine serum (Cat. No. 10091148, Gibco, Waltham, MA, United States) and 1% penicillin/streptomycin (Cat. No. 60162ES76, Yeasen, Shanghai, China) at 37°C and 5% CO_2_. The IR cell model was prepared by treating with 1 μM dexamethasone (Dex) (D1756, Sigma-Aldrich, St. Louis, MO, United States), 300 μM palmitic acid (PA) (T2908, TargetMol, Massachusetts, United States), and 30 mM 2-deoxy-D-ribose (dRib) (Sangon Biotech, Shanghai, China), for 24 h, respectively. Then IR-Hepa1-6 cells were treated with 24 h of 5 μg/ml promethazine (HY-B0781, MedChemExpress).

### 2.9 Lenti-shRNA construction and virus infection with Hepa1-6

The two pairs of short hairpin RNA (shRNA) sequences of mouse ubd are shown in Supplementary Table S1. A scrambled sequence was used as a shRNA control. And then the virus packaging and collection of viruses. Hepa1-6 was infected with lenti-shRNA using polybrene. After infection for 48 h, cells were screened with puromycin for subsequent experiments.

### 2.10 Quantitative reverse transcription polymerase chain reaction (qRT-PCR)

Total RNA extraction and qRT-PCR were performed using Trizol (Sigma-Aldrich, St. Louis, MO, United States), the Primescript™ RT Master Mix kit (Takara, Shiga, Japan) and SuperReal Premix plus (SYBR green) kit (Tiangen Biotech, Beijing, China) according to manufacture instruction. Data were analyzed using the 2^−ΔΔCt^ method (21). Beta-actin was used as an internal control. The primer sequences are shown in Supplementary Table S2.

### 2.11 Western blotting

Hepa1-6 cells and four ISTs were lysed in radioimmunoprecipitation assay buffer (Cat. No. P0013B, Beyotime, Jiangsu, China) with protease inhibitors (Thermo Fisher Scientific, Waltham, MA, United States) to extract whole-cell proteins. Protein concentration was quantified using a bicinchoninic acid kit (Cat. No. 20201ES76, Yeasen, Shanghai, China). A sample (20 μg) of each protein was then separated by sodium dodecyl sulfate–polyacrylamide gel electrophoresis, transferred to a polyvinylidene fluoride membrane (Millipore, Burlington, MA, United States), and blocked with 5% bovine serum albumin at room temperature for 1 h. After incubation overnight at 4°C with primary antibodies (Supplementary Table S3), membranes were incubated with a secondary antibody for 2 h. The secondary antibody used was horseradish peroxidase-conjugated goat anti-rabbit IgG (1:10,000; Cat. No. SA00001-2, Proteintech, Rosemont, IL, United States). The proteins were visualized using enhanced chemiluminescence (Cat. No. E412-02, Vazyme, Jiangsu, China). Tanon 5200S (Tanon Science & Technology, Shanghai, China) was used to evaluate the density of protein bands, and relative protein levels were quantified using ImageJ software.

### 2.12 Statistical analysis

Results were presented as the mean ± Standard Deviation (SD). The student’s *t*-test was used for the statistical significance analysis. A *p*-value <0.05 was considered to be statistically significant.

## 3 Results

### 3.1 Flowchart of this study

**Figure 1:**
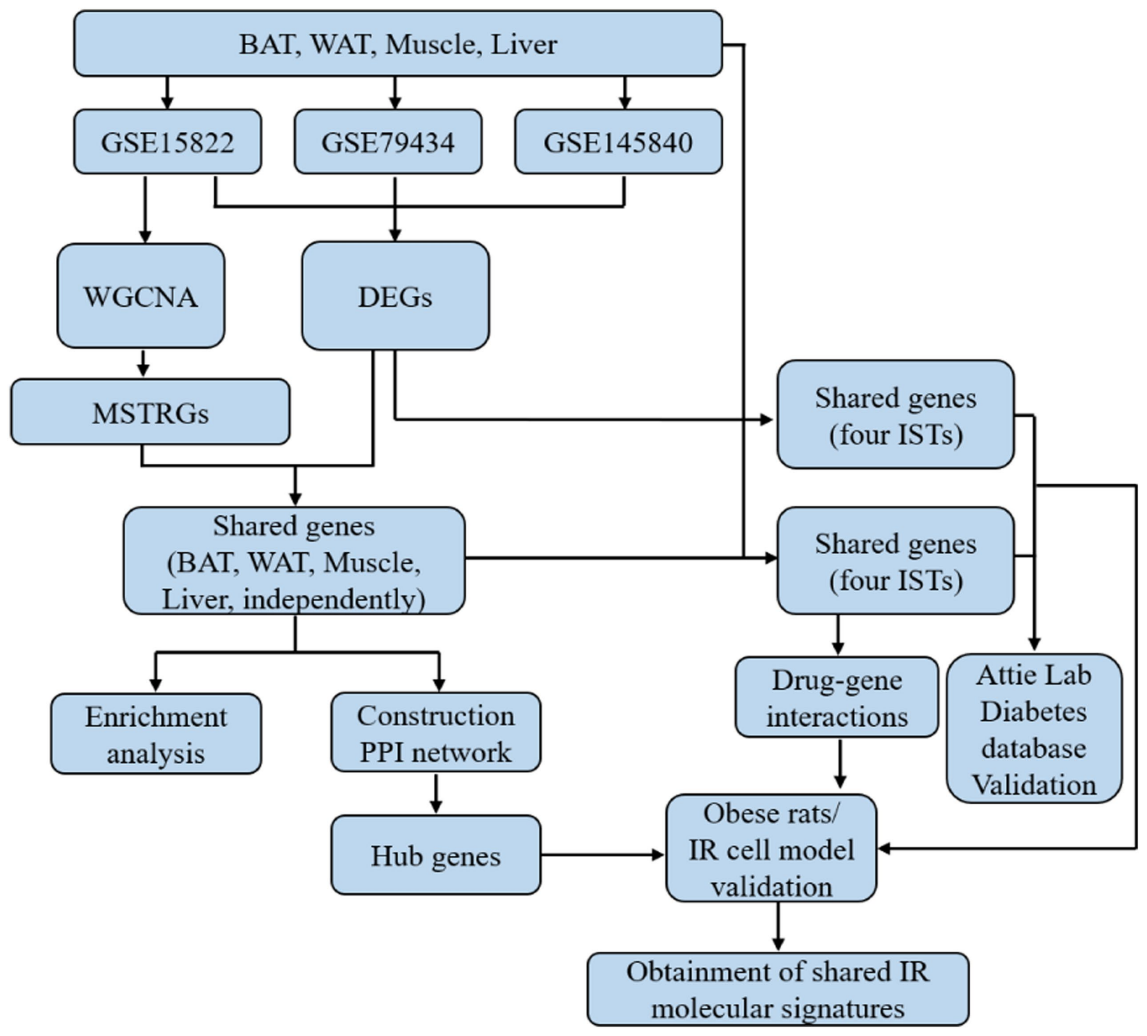
The flowchart of this study.

The overall workflow of this study is shown in Figure 1.

### 3.2 Obtainment of DEGs in BAT, WAT, muscle, and liver

Three GEO data sets (GSE15822, GSE79434, and GSE145840) were enrolled in this study. The details of the datasets are shown in Table 1. After removing the batch effects, DEGs were obtained based on adjusted *p*-value <0.05 and |logFC| > 1. The information on DEGs is demonstrated in Table 2. Muscle changes were less pronounced, with the largest number of DEGs in two adipose tissues, followed by liver. The heatmap demonstrated the top 50 upregulated and downregulated DEGs in each type of tissue (Supplementary Figure S1).

**Table 1:** The detailed information of the three datasets used in Meta-analysis.

Dataset	Platform	Species	Samples	Models	Experimental group	Control group	Reference
GSE15822	Illumina mouse-6 v1.1 expression bead chip	Mouse	BAT, WAT, Muscle, Liver	45 kcal% fat for 6 weeks	6	6	PMID:20215417
GSE79434	Affymetrix Mouse Gene 1.0 ST Array [transcript (gene) version]	Mouse	BAT, WAT, Muscle	60 kcal% fat for 8 weeks	4	4	PMID: 27166587
			Liver		5	5	
GSE145840	HiSeq X Ten (*Mus musculus*)	Mouse	BAT	60 kcal% fat for 14 weeks	3	3	PMID: 32780721
			Muscle		4	3	
			WAT, Liver		4	4	

**Table 2:** The amount of differentially expressed genes (DEGs) between HFD and NCD.

	Total	Up	Down
BAT	3,365	1807	1,558
WAT	3,950	2,139	1811
Muscle	134	74	60
Liver	1,652	1,020	632

### 3.3 WGCNA analysis

**Figure 2:**
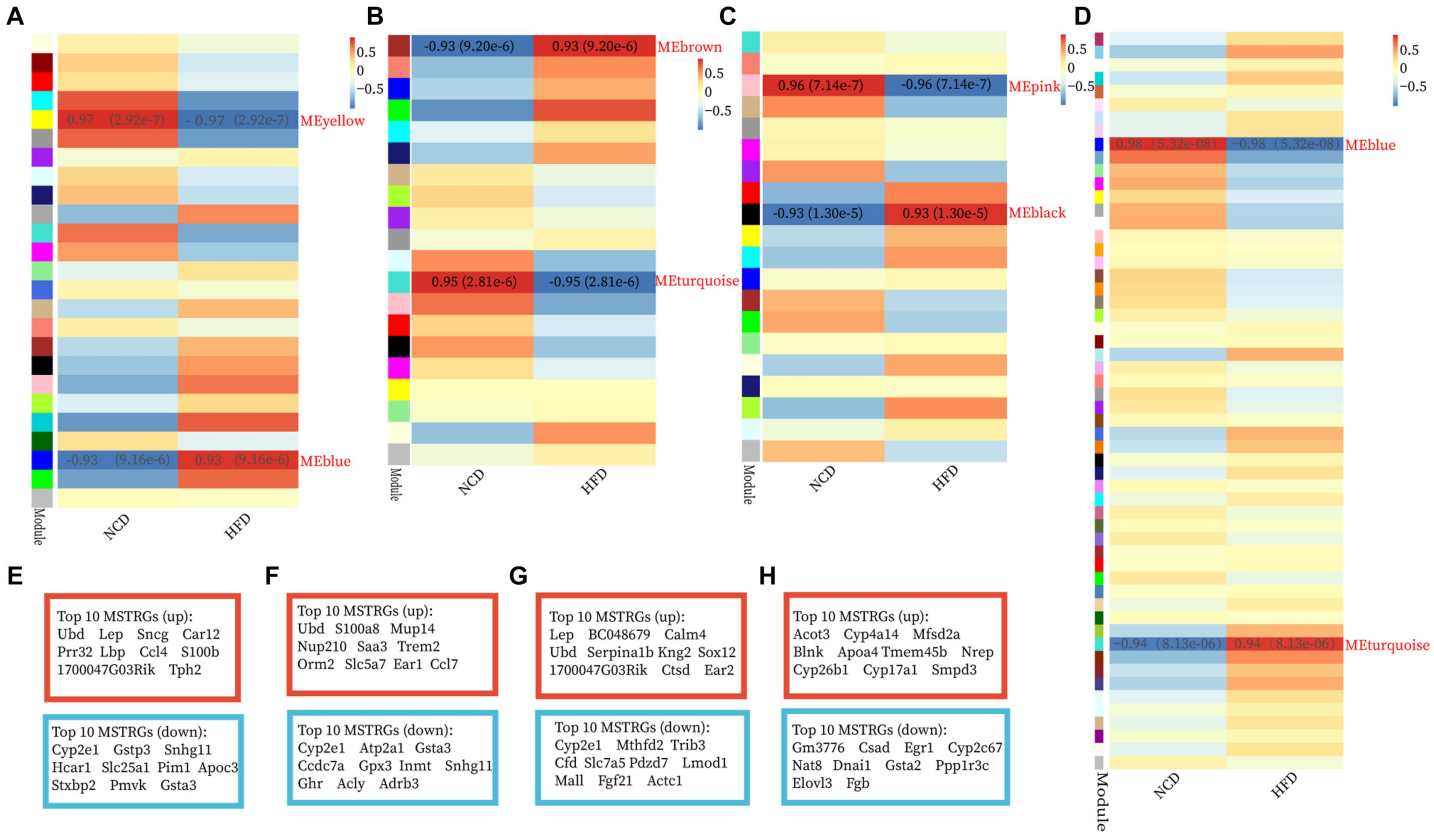
Construction of WGCNA modules and module enrichment analyses. **(A–H)** The heatmap of the module-trait relationships and the display of the top 10 upregulated and downregulated MSTRGs of BAT **(A,E)**, WAT **(B,F)**, Muscle **(C,G)**, and Liver **(D,H)**.

The GSE15822 dataset with the large sample size was used as the test set while the other two datasets as validation sets. The top 5,000 greatest variance genes in the GSE15822 dataset were used to identify co-expression modules. These genes were clustered into multi-co-expression modules as shown in Supplementary Figures S2A–D. Afterward, the correlations between the module and clinical traits were calculated. In BAT, the blue module had the strongest positive relation with HFD (cor = 0.93; *p* = 9.16 e−6), while the yellow module had the strongest negative relation with HFD (cor = −0.97; *p* = 2.92 e−7) (Figure 2A). For WAT, the brown module showed the strongest positive correlation with HFD (cor = 0.93; *p* = 9.20 e−6), and turquoise had the strongest negative correlation with HFD (cor = −0.95; *p* = 2.81 e−6) (Figure 2B). The black module demonstrated the highest positive correlation with HFD (cor = 0.93; *p* = 1.30 e−5), and the pink module had the highest negative correlation with HFD (cor = −0.96; *p* = 7.14 e−7) in muscle (Figure 2C). In liver, the turquoise module had the strongest positive relation with HFD (cor = 0.94; *p* = 8.13 e−6), while the blue module had the strongest negative relation with HFD (cor = −0.98; *p* = 5.32 e−8) (Figure 2D). In addition, the MSTRGs was used as important module genes for subsequent analysis. The top 10 upregulated and downregulated MSTRGs were also shown in boxes in Figure 2, Ubd (up) was listed among the top 10 genes in four ISTs and was proposed to have a central role in IR.

GO-BP and KEGG enrichment analyses of the “MSTRGs” were performed. Regardless of these four tissues, the enrichment results were common in metabolism-related pathways and oxidation–reduction process (Supplementary Figures S2E–H). In two adipose tissues, the up-regulated MSTRGs were most enriched in immune and inflammatory-related processes (GO-BP), immune and inflammatory-related diseases (KEGG pathway), while many metabolic process (GO-BP), metabolic pathways (KEGG pathway) were enriched from the down-regulated MSTRGs. Furthermore, WAT showed an effect on the development and contraction of skeletal muscle (Supplementary Figures S2E,F). In addition to being enriched in many metabolic processes, muscle was also mainly enriched in regulation of transcription by RNA polymerase II (Supplementary Figure S2G). In liver, the strongest positively (up) correlated module genes were mainly enriched in cholesterol/steroid/fatty acid biosynthetic (GO-BP), process biosynthesis of unsaturated fatty acids (KEGG pathway) (Supplementary Figure S2H).

### 3.4 Identification of shared genes between MSTRGs and DEGs, and enrichment analysis

**Figure 3:**
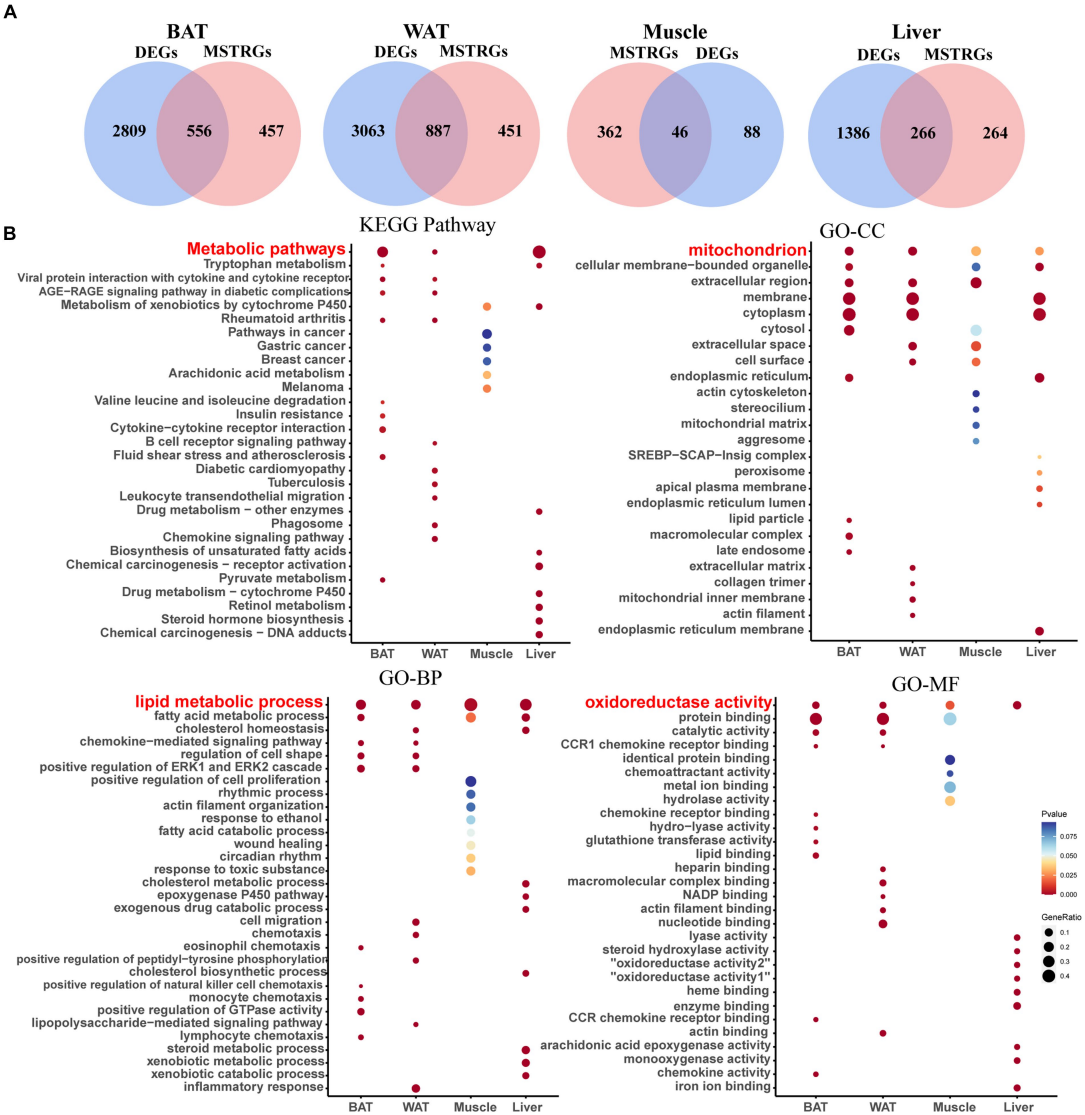
Screening of shared genes and Enrichment Analysis. **(A)** Venn diagram for intersections between DEGs and MSTRGs. **(B)** Go and KEGG pathways enriched in the above-shared genes.

Shared genes were obtained between DEGs and MSTRGs in four ISTs (Figure 3A). The GO and KEGG enrichment analyses were performed with these shared genes for individual tissues (Figure 3B).

As shown in Figure 3A, BAT and WAT tissues had the largest number of shared genes between NCD- and HFD-exposed mice, followed by liver. In contrast, there was the smallest number of shared genes in skeletal muscle, which was consistent with previous reports (22). Figure 3B displays the top 10 terms of both GO and KEGG analyses across four ISTs, respectively. The shared terms across ISTs were found, including lipid metabolic process (biological process, BP), mitochondrion (cellular component, CC), oxidoreductase activity (molecular function, MF), and metabolic process (Kyoto Encyclopedia of Genes and Genomes, KEGG), which encouraged us to identify the shared key molecular signatures in IR across four ISTs.

### 3.5 PPI network construction and hub genes recognition and verification

The PPI network was constructed in STRING (Supplementary Figures S3A–D). To identify the hub genes in the shared parts between DEGs and MSTRGs, Cytohubba was performed. All the gene codes and edges were calculated, and the hub genes were filtered using the MCC algorithm. The top 20 hub genes in BAT, WAT, and liver, and the top 10 genes in muscle were identified as hub genes (Supplementary Figures S3E–H). Detailed information about the top 20/10 hub genes are shown in Table 3 and Supplementary Table S4. In two adipose tissues, the top 20 hub genes belonged to immune and inflammatory-related factors, of which Gpr183, Tas2r104 (BAT) and Gng7, P2ry13 (WAT) were novel to IR. All of the top 10 hub genes in muscle had been reported to be involved in IR before. In liver, these genes (Cyp2c39, Cyp2b13, Aox3, Cyp4a31, Ugt2b1, Ugt2b35, and Por) were mainly involved in cytochrome P450 metabolism, and none has been reported in IR. The top 10 betweenness centrality (BC) genes in BAT, WAT, muscle and liver were identified (Supplementary Table S5). Rem1, Actc1 and Ugt2b1 were novel to IR. Importantly, we also found shared genes between BC genes and hub genes, namely, App in BAT, Vegfa, App in WAT, Lep, Decr1, Cdh1, Cyp2e1, Acadl, Clu, Fgf21, Cfd, Plin5 in muscle, Ugt2b1 in liver, respectively. Among them, only Ugt2b1 with high BC was a novel hub gene to IR.

**Table 3:** The top hub genes, BC genes and share genes of the two are found in the PPI network in each type of tissue.

Tissue	Published role in IR	Hub genes	BC genes	Shared genes between hub genes and BC genes
BAT	Known	Ccl4, Cxcl12, Cxcl16, Cxcl1, Ccl5, App, Anxa1, Sst, Aplnr, Hcar1, Hcar2, Emr1, Itgax, Csf1r, Ctss, Cd53, Ly86, Mpeg1	Cdk1, Decr1, Ccnd1, App, Lep, Grb2, Ccl2, Ctla4, Cdh5, Bmp4	App
	Unknown	Gpr183, Tas2r104	—	—
WAT	Known	App, Cxcl1, Ccl4, Cxcl9, Ccr7, Ccr5, Cxcl12, Ccl5, Agt, Sucnr1, Cnr2, Ccl6, Hcar1, Hcar2, Fermt3, Vegfa, Orm2, Serpina3n	Cd44, Apob, Vegfa, Mmp9, App, Ccnb1, Rab7, H6pd, Tlr2	Vegfa, App
	Unknown	Gng7, P2ry13	Rem1	—
Muscle	Known	Lep, Cyp2e1, Decr1, Cfd, Cdh1, Clu, Acadl, Ephx1, Fgf21, Plin5	Lep, Decr1, Cdh1, Cyp2e1, Acadl, Clu, Fgf21, Cfd, Plin5	Lep, Decr1, Cdh1, Cyp2e1, Acadl, Clu, Fgf21, Cfd, Plin5
	Unknown	—	Actc1	—
Liver	Known	Cyp1a2, Cyp2c70, Cyp2b9, Cyp3a11, Cyp4a14, Aox1, Gsta2, Gstm4, Gstm1, Gstm6, Ephx1, Cyp17a1, Abcb11	Egfr, Apoa4, Pcsk9, Hspa5, C8b, Nr1d1, G6pc, Cyp2c70, Gamt	—
	Unknown	Cyp2c39, Cyp2b13, Aox3, Cyp4a31, Ugt2b1, Ugt2b35, Por	Ugt2b1	Ugt2b1

**Figure 4:**
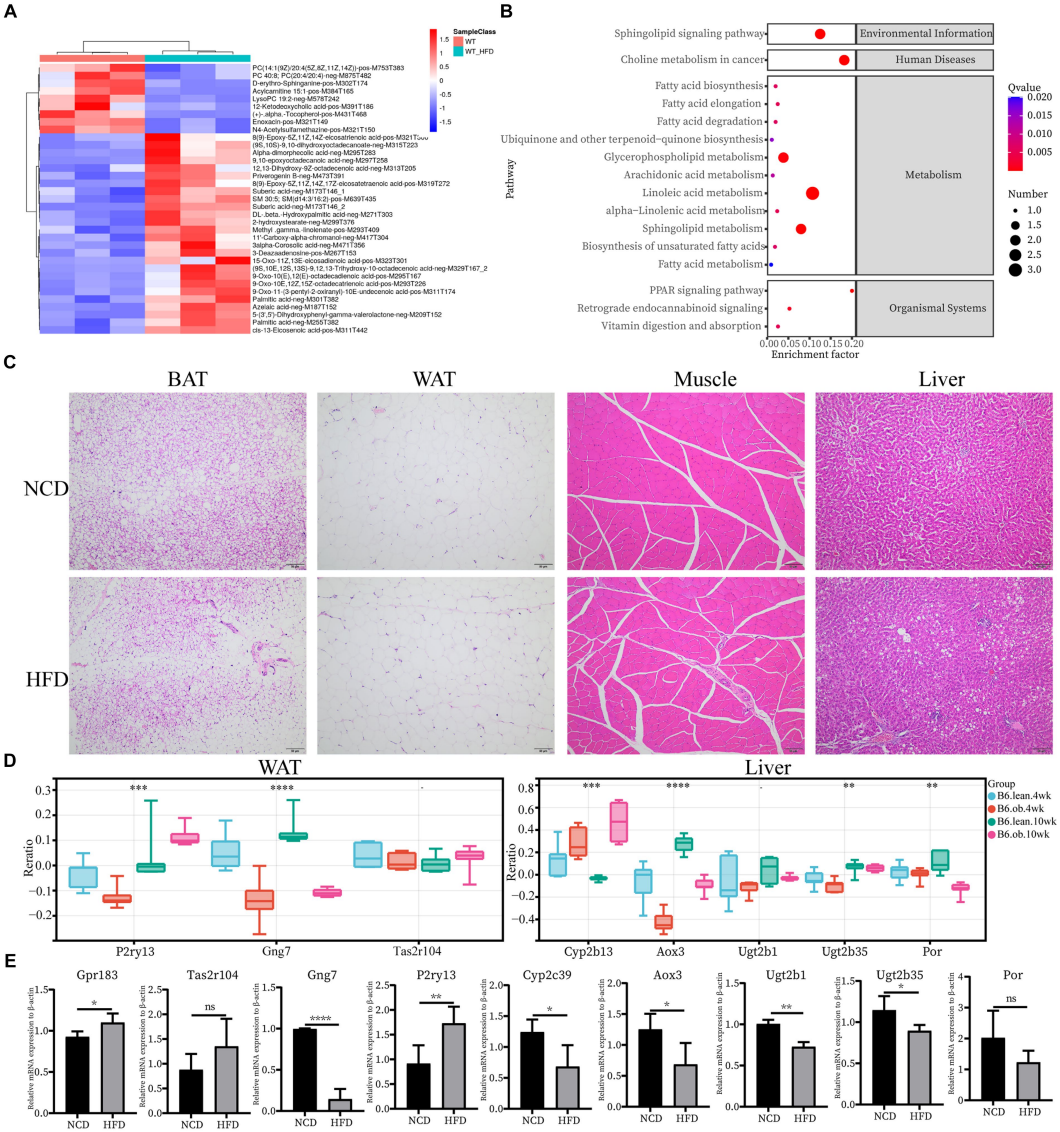
Confirmation of hub genes of interest at mRNA level. **(A)** Clustering heatmap analysis of the main differentially abundant metabolites in WT vs. WT-HFD. **(B)** Pathway enrichment analysis of differentially abundant metabolites in WT vs. WT-HFD. **(C)** Hematoxylin and eosin (H&E) staining of BAT, WAT, Muscle, and Liver; scale bar, 50 um. **(D)** Expression changes of interested hub genes (Pr2y13, Gng7, and Tas2r104 in AT and Cyp2b13, Aox3, Ugt2b1, Ugt2b35, and Por in Liver) between obese and lean B6 mice were validated in the Attie Lab Diabetes database. **(E)** qPCR analysis of Gpr183 and Tas2r104 in BAT, Gng7 and P2ry13 in WAT, and Cyp2c39, Aox3, Ugt2b1, Ugt2b35 and Por in Liver.

Next, we confirmed the expression pattern of the hub genes of interest in 12-week HFD-fed rats. First, plasma metabolomics and morphological changes after HFD feeding were analyzed to ensure the successful construction of the HFD-induced obesity model. Metabolomics analysis revealed significantly elevated levels of medium and long-chain fatty acids (FA) in plasma in the HFD group (Figure 4A). The HFD resulted in significant changes in signaling pathways, for example, FA metabolism and FA biosynthesis (Figure 4B). The significantly enlarged size of adipocytes and fat accumulation in the liver after HFD were observed (Figure 4C). All of the above supported that the HFD-induced obesity model was successful. Since the top 10 hub genes in skeletal muscle have been reported in IR, the expression of the novel hub genes in other ISTs was verified with the Attie Lab Diabetes database (Figure 4D). Experimentally, the expression patterns of the novel hub genes in our obesity rats were consistent with the findings of the meta-analysis (Figure 4E).

### 3.6 Identification and validation of shared IR molecular signatures and their drugs across four ISTs

**Figure 5:**
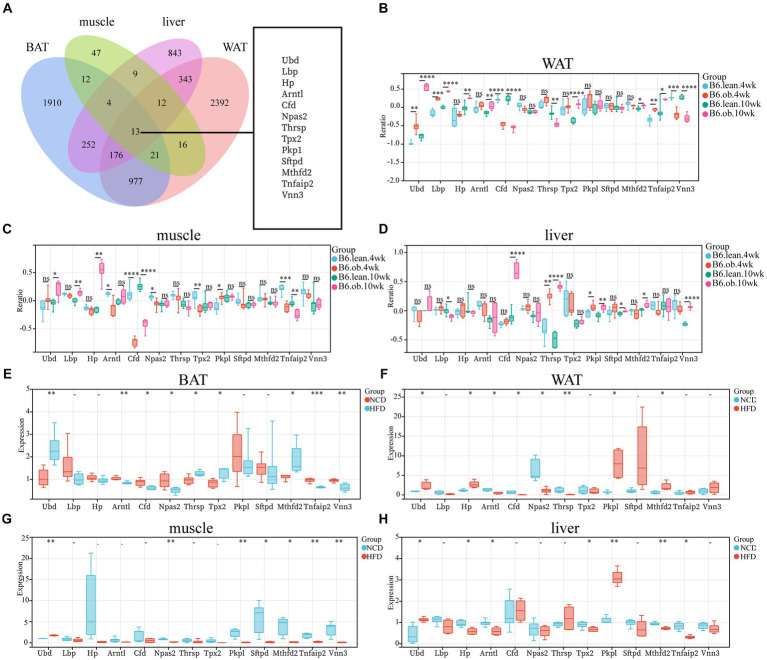
Identification and Validation of the Shared DEGs in four ISTs. **(A)** Venn diagram for an overlap of DEGs between BAT, WAT, Muscle, and Liver. **(B–D)** Expression changes of 13 common genes in ISTs between obese and lean B6 mice were validated in the Attie Lab Diabetes database. **(E–H)** qPCR analysis of 13 common genes in ISTs in NCD vs. HFD. Significance was set as *p < 0.05; **p  < 0.01; ***p  < 0.001 or ****p  < 0.0001.

The overlap of DEGs in four ISTs was observed between HFD and NCD as a total of 13 genes (Ubd, Lbp, Hp, Arntl, Cfd, Npas2, Thrsp., Tpx2, Pkp1, Sftpd, Mthfd2, Tnfaip2, and Vnn3) (Figure 5A). The detailed information for 13 shared genes is shown in Table 4. Most of these genes (Ubd, Hp, Arntl, Npas2, Tpx2, Pkp1, and Vnn3) were consistently regulated across four ISTs, while Lbp, Cfd, Thrsp., Sftpd, and Vnn3 were only oppositely regulated in liver compared to the others. Mthfd2 was upregulated in adipose tissues and downregulated in muscle and liver. Tnfaip2 was only oppositely regulated in WAT compared to the others. Among them, six genes (Ubd, Tpx2, Pkp1, Mthfd2, Tnfaip2, and Vnn3) have never been reported in IR, and Npas2, Thrsp., Sftpd have been reported in previous IR-related studies lacking mechanistic study (23–25).

**Table 4:** Shared DEGs found in meta-analysis.

Name	Published role in IR	Functions in IR	Regulation in four tissues			
			BAT	WAT	Muscle	Liver
Lbp	PMID: 22184060	Circulating lipopolysaccharide-binding protein (LBP) as a marker of obesity-related IR	Up	Up	Up	Down
Hp	PMID: 21873550	Haptoglobin (Hp) is upregulated in both inflammation and obesity	Up	Up	Up	Up
Arntl	PMID: 35238641	Disruption of the circadian clock component BMAL1 elicits an endocrine adaption impacting on insulin sensitivity and liver disease	Down	Down	Down	Down
Cfd	PMID: 31700183	The adipokine adipsin/complement factor D controls the alternative complement pathway and generation of complement component C3a, which acts to augment beta cell insulin secretion	Down	Down	Down	Up
Npas2	PMID: 33602006	A circadian gene, associated with IR parameters	Down	Down	Down	Down
Thrsp	PMID: 35715726	Insulin-inducible THRSP maintains mitochondrial function and regulates sphingolipid metabolism in human adipocytes	Down	Down	Down	Up
Sftpd	PMID: 26568332	Implicated in diabetes	Up	Up	Up	Down
Ubd	N/A	N/A	Up	Up	Up	Up
Tpx2	N/A	N/A	Up	Up	Up	Up
Pkp1	N/A	N/A	Up	Up	Up	Up
Mthfd2	N/A	N/A	Up	Up	Down	Down
Tnfaip2	N/A	N/A	Down	Up	Down	Down
Vnn3	N/A	N/A	Down	Down	Down	Up

To validate the expression patterns of 13 shared genes of IR from meta-analysis, the Attie Lab Diabetes database and HFD-fed rats were used, respectively. We found that the expression pattern of 13 shared genes with the 4 or 10-week B6 obese diabetic mice was consistent with meta-analysis (Figures 5B–D). Also, the same expression patterns of 13 shared genes in four ISTs from HFD-obesity rats by RT-qPCR were found (Figures 5E–H).

**Figure 6:**
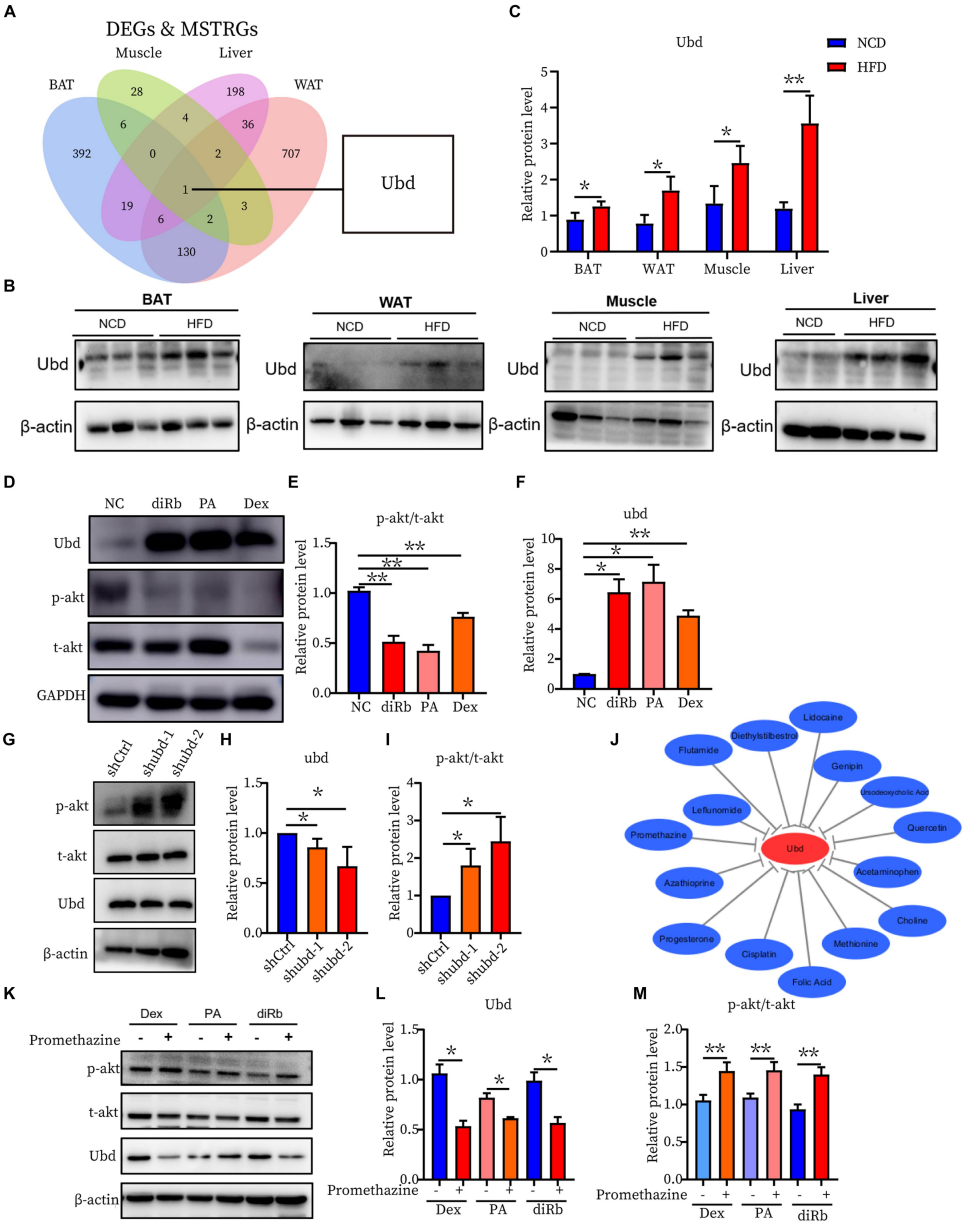
Identification and Validation of the shared IR molecular signatures and their drugs across four ISTs. **(A)** Venn diagram for an overlap of intersections between DEGs and MSTRGs between BAT, WAT, Muscle, and Liver. **(B,C)** Western blot analysis of the protein expression of Ubd in BAT, WAT, muscle, and liver in rats on NCD and HFD for 12 weeks. **(D–F)** Western blot analysis of the protein expression of p-akt/t-akt and Ubd in Hepa1-6 cells with the treatment 24 h of 1 μM Dex, 300 μM PA, and 30 mM dRib, respectively. **(G–I)** Western blot analysis of insulin signaling pathway p-akt/akt expression after Ubd knockdown in liver Hepa1-6 cells. **(J)** Drug-gene interactions network with lowing Ubd expression drugs and Ubd gene using the CTD database. The grey arrows represent that the medicinal drugs will decrease the expression of the Ubd gene. **(K–M)** Western blot analysis of the protein expression of p-akt/t-akt and Ubd in IR-Hepa1-6 cells with the treatment of 24 h of 5 μg/ml promethazine. Significance was set as *p  < 0.05; **p  < 0.01.

To further investigate the correlation of the IR trait with 13 DEGs, the shared genes between DEGs and MSTRGs from WGCNA were selected. Only one overlapping gene Ubd was obtained (Figure 6A). Western blot showed Ubd expressed higher in liver than other ISTs in obese rats (Figures 6B,C). Since significant upregulation of Ubd expression in liver of obese rats, we used hepatocytes line Hepa1-6 for subsequent *in vitro* experiments. We established an IR cell model by treating cells with dexamethasone (Dex), palmitic acid (PA), and 2-deoxy-d-ribose (dRib), for 24 h, respectively and the results showed that the expression of UBD was significantly elevated in IR-Hepa1-6 cells (Figures 6D–F). Considering that the proximal segment of the insulin signaling pathway was conserved and Ubd was considered to be tightly correlated with IR trait across four ISTs, we speculated that Ubd may target the proximal segment of insulin signaling and affect p-Akt level. To this end, knockdown of Ubd in Hepa1-6 cells was performed. We found that the p-akt/t-akt ratio was significantly increased after the knockdown of Ubd (Figures 6G–I).

Finally, we used the CTD database to construct a medical drug-gene interaction network in which various drugs can reduce the mRNA expression levels of the Ubd gene (Figure 6J). We found that all drugs that downregulated Ubd expression improved IR except PMZ (Table 5). We focused on the PMZ. PMZ did reduce the expression of Ubd and upregulated p-akt/t-akt ratio in the IR cell model (Figures 6K–M).

**Table 5:** List of drug-gene interactions with medicinal drugs (down-regulated the expression of Ubd) and Ubd gene using the CTD database.

Drug	Published role in IR	Functions in IR
Choline	PMID: 27908547	High dietary choline and betaine intake is associated with low IR in the Newfoundland population
Acetaminophen	PMID: 20065957	Abolished oxidative stress in WAT, improved glucose tolerance and IR
Azathioprine	PMID: 1478380	Immunotherapy with cyclosporin A and azathioprine can slow disease progression(Severe Type B IR)
Flutamide	PMID: 12788862	Low-dose flutamide-metformin therapy reverses IR (PCOS)
Lidocaine	PMID: 27168847	Neurolytic celiac plexus block by 0.5% lidocaine, enhanced skeletal muscle insulin signaling and attenuates IR in GK rats
Leflunomide	PMID: 29496905	Leflunomide treatment normalized blood glucose levels and overcame IR in glucose and insulin tolerance tests in ob/ob and HFD-fed mice
Promethazine	N/A	N/A
Progesterone	PMID: 18657616	Progesterone protected from IR in STZ-induced diabetic rats
Methionine	PMID: 34700006	Dietary supplementation of methionine improves hepatic steatosis, IR, inflammation, fibrosis, and bone health.
Ursodeoxycholic Acid	PMID: 29093351	UDCA may have protective effects against palmitate-induced decreases in responsiveness to insulin
Cisplatin	PMID:29039558	IR increased cisplatin resistance; Insulin combined with cisplatin promoted apoptosis of cancer cells
Diethylstilbestrol	PMID: 15846301	IGFBP-6 gene expression and protein levels significantly increased after DES treatment
Quercetin	PMID: 34101925	Reduction of IR
Folic Acid	PMID: 29986310	Folic acid (FA) supplementation may protect from obesity and IR
Genipin	PMID: 29377296	Genipin ameliorates diet-induced obesity by promoting lipid mobilization and browning of white adipose tissue in rats

## 4 Discussion

**Figure 7:**
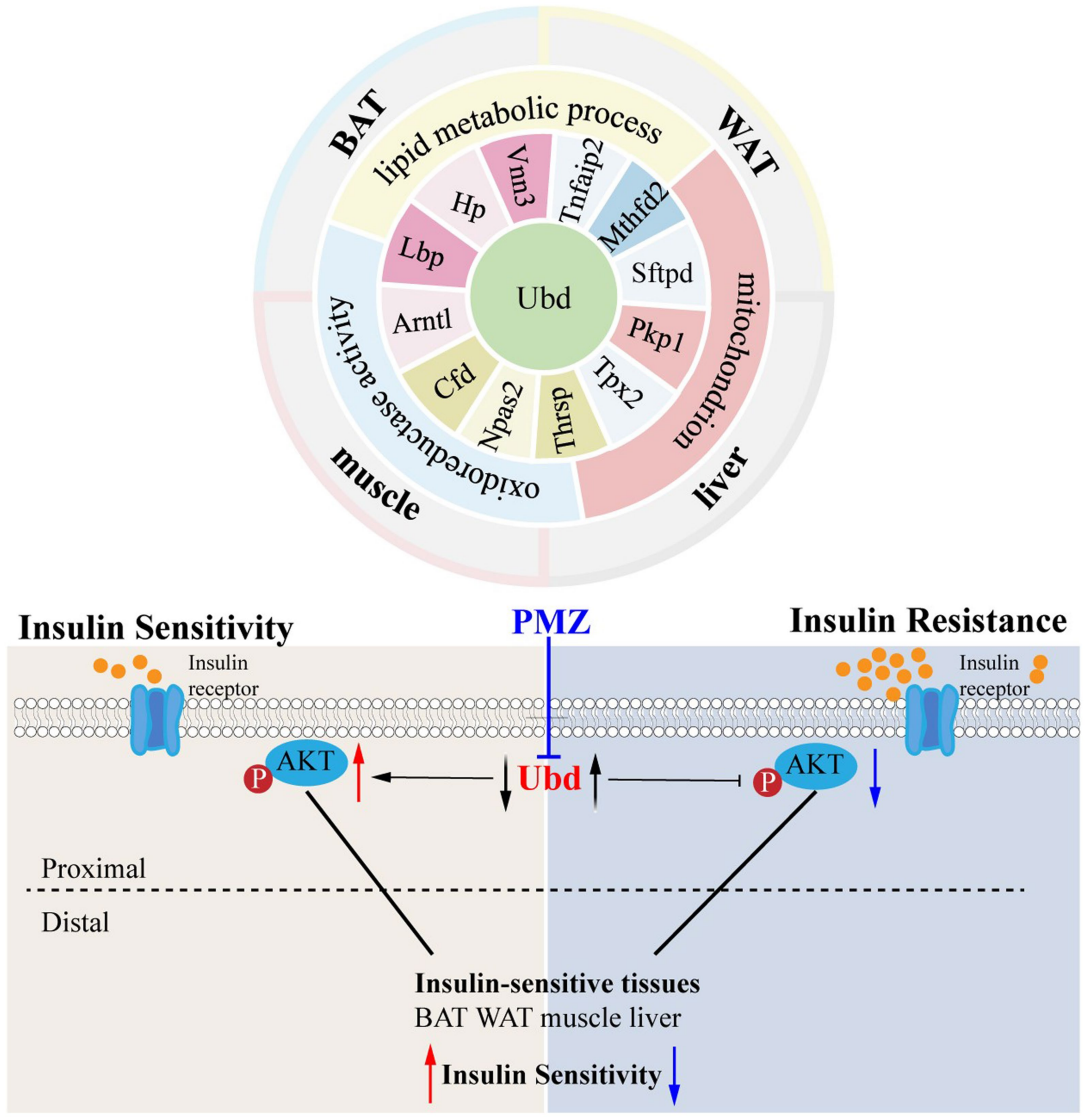
Schematic molecular mechanism and the main conclusion of this study.

The ISTs bear distinct IR phenotypes due to distal metabolic features and distal segments of the insulin signaling pathway. In this present study, using the hypothesis-driven approach, we performed suitable step-by-step bioinformatics analysis combining WGCNA analysis to obtain and validate the 13 shared IR molecular signatures across 4 ISTs. Among them, UBD was identified to be highly correlated with IR trait. The drug PMZ improved IR by downregulation of UBD level in Hepa1-6. Our work will provide a novel therapeutic target and drug for IR treatment and a useful clue to translational research in the IR field. A working model is proposed in Figure 7.

Since IR is a systemic disorder, as expected, there were common KEGG and GO terms enriched across four ISTs in obese mice. Under an obese context, several known terms of KEGG pathways related to metabolic process, GO terms involved in lipid metabolic process (BP), mitochondrion (CC), and oxidoreductase activity (MF) were enriched across four ISTs (Figure 3B). Accordingly, 13 shared genes (Ubd, Lbp, Hp, Arntl, Cfd, Npas2, Thrsp., Tpx2, Pkp1, Sftpd, Mthfd2, Tnfaip2, and Vnn3) of DEGs across four ISTs were obtained between HFD and NCD (Figure 5A). Compared with the meta-analysis, among 13 molecular signatures for IR, Ubd, Hp, Tpx2, and Pkp1 were upregulated while Arntl and Npas2 were downregulated in four ISTs in obese rats. And the expression pattern of other Lbp, Cfd, Thrsp., Sftpd, and Vnn3 was only oppositely regulated in liver compared to the others (Table 4), because pathways in the liver were often regulated in the opposite direction from adipose tissues, reflecting the overall flow of energy in HFD conditions (22). Among them, some shared DEGs (Ubd, Tpx2, Pkp1, Mthfd2, Tnfaip2, and Vnn3) have not been reported before in IR-relevant studies. These DEGs with consistent expression patterns across four ISTs will be potential targets by gene manipulation to improve systemic IR. So novel molecular signatures of IR (UBD, Tpx2, and Pkp1) with consistent expression patterns across four ISTs were discussed below.

Tpx2, short for targeting protein for xklp2, was a microtubule-associated microtubule nucleation factor that was required for mitotic spindle function. Tpx2 mediates AURKA localization to spindle microtubules (26) while TPX2 is inactivated upon binding to importin-alpha (27). Pkp1 (Plakophilin 1) was a member of the arm-repeat (armadillo) and plakophilin gene families. CRISPR-Cas9 PKP1 knockout severely impaired cell proliferation, and increased cell dissemination (28). The high expression of PKP1 facilitated cancer cells to form clusters in circulation and also activated the PI3K/AKT/Bcl–2–mediated pathway to increase cell survival (29). Our study showed that the expression of Tpx2 and Pkp1 was elevated in four ISTs in the presence of IR, how they regulated insulin signaling pathway deserves to be explored in depth.

Ubd was the only one that was identified from the shared parts between DEGs and MSTRGs, indicating its strong correlation with the IR trait (Figure 6A). Ubd is a ubiquitin-like protein modifier. Strong synergistic upregulation of Ubd mRNA and protein was observed upon exposure to the interferon-γ and tumor necrosis factor (30, 31). Besides Ubd directly targets its substrate for degradation by the 26S proteasome (32). Although Ubd is a gene responsive to stress and inflammation (33) and is already thought to play a role in inflammation driven diseases (34), we cannot draw a conclusion that any gene responsive to stress and inflammation is a candidate for insulin resistance. By our validation experiment, either overexpression or knockdown, we clarified the direct negative correlation between UBD expression and p-akt level, a major insulin resistance index. In the present study, we found all drugs that downregulated Ubd expression improved IR (Table 5), and the knockdown of Ubd significantly promoted the p-akt/akt ratio in Hepa1-6 cells (Figures 6G–I). Whether the p-akt regulation by UBD is direct or indirect remains unknown.

Recent studies have shown that hyperinsulinemia and associated IR promote cell senescence (CS) in both human adipose and liver cells. Conversely, increased CS promotes cellular IR, showing their interdependence. Hallmarks of aging are functionally intertwined and drive the pathophysiology of many chronic disorders, affecting tissues directly involved in IR development (35, 36). Very recently, Ubd expression was observed in human proximal tubular cells *in vitro* and during aging (37). A novel role of Ubd in immune metabolic regulation that impact aging and chronic disease has been revealed, Ubd KO extended lifespan and enhanced insulin sensitivity in skeletal muscle tissues (38). Thus, IR-related markers are potential targets for aging and vice versa. Ubd will be a novel therapeutic target for ameliorating IR and aging progression.

The PPIs in cells form a complicated network and have a significant role in physiological and pathological processes. Tissue-specific PPI network was constructed, and hub genes were predicted and further verified from a single IST (Supplementary Figure S3 and Figure 4). However, there was no shared hub gene enriched across four ISTs, supporting distinct metabolic features in four ISTs. Some tissue-specific hub genes, such as Gpr183, Tas2r104 (BAT), Gng7, P2ry13 (WAT), and Cyp2c39, Cyp2b13, Aox3, Cyp4a31, Ugt2b1, Ugt2b35, Por (Liver) (Table 3), have not yet been reported in IR, which will be very helpful to understanding the tissue-specific IR. In BAT, Tas2r104 was identified as a novel hub gene in IR. Tas2r104 belongs to the superfamily of G protein-coupled receptors (GPCRs) for bitter sensing (39). Bitter taste receptors (T2R) are expressed in the gastrointestinal tract, and dietary stimuli can modulate taste receptor expression and modulate tastemaker status, thereby feeding back to regulate preference and intake. The unbalance of T2R can increase the risk of metabolic disease (40). In our study, we found Tas2r104 expression was slightly upregulated in HFD-rats. We speculated that up-regulation of Tas2r104 may alter food intake and preference, participating in the development of IR. Its real function needs to be further investigation. In WAT, Gng7 and P2ry13 were first reported as hub genes in IR in our study. Gng7 is involved as an olfactory receptor downstream signaling molecule, the its specific role is unknown (41). P2ry13, purinergic receptor P2Y, is a G protein-coupled metabolic receptor. The down-regulated Gng7 and up-regulated P2ry13 were detected in HFD-rat. How they participate in IR remains elusive. In liver, three types of drug metabolizing enzymes (DMEs) were identified as novel hub genes, including oxidases (Aox3and Por), Cytochrome P450s (Cyp2c39, Cyp2b13, and Cyp4a31) and UDP-glucuronosyltransferases (Ugt2b1 and Ugt2b35). In particular, Ugt2b1 was also identified as high BC gene (Table 3). After our validation, except no significant difference of Por between NCD and HFD group, the others showed decreased expression in HFD-rat (Figure 4E). It is well known that DMEs can reduce the formation of toxic metabolites to mitigate toxicity (42). How above mentioned DMEs affect the development of IR deserve further exploration.

Protein tyrosine phosphatase-1B (PTP1B) is a negative regulator of the insulin signaling pathway. PTP1B inhibits insulin action by dephosphorylating insulin receptor β subunit, a proximal component of the insulin signaling pathway. Reduction of PTP1B has been shown to be effective against IR (43). PTP1B inhibition has been a potential key therapeutic target against IR. However, the development of PTP1B inhibitors has been hindered by two factors: the permeability of the cell membrane of PTP1B inhibitors and the selectivity of PTP1B inhibitors (44). The identified candidate UBD in the present study also affected the proximal component of the insulin signaling pathway, p-Akt.

Currently, the main drugs to improve insulin resistance were thiazolidinediones (TZD) (pioglitazone) and PPAR full agonists (selegiline sodium) and others, but they have been associated with side effects including sodium retention, edema, congestive heart failure, and damage liver and kidney functions (45, 46). Therefore, it is important to find new and effective drugs to improve insulin resistance. Promethazine hydrochloride is a first-generation H1 receptor antagonist, antihistamine, and antiemetic medication that can also have strong sedative effects (47). In our study, a new use for PMZ was revealed. PMZ inhibited UBD expression and increased p-akt level in the IR cell model in Hepa1-6. Either direct intervention of UBD expression or administration of PMZ merit further clinical investigation for IR treatment.

However, the present study has several limitations that need to be addressed. First, a specific knock-in/out mouse model will provide a better understanding of the underlying mechanisms involving UBD during IR. Second, treatment of IR mice with the drug PMZ will visualize the therapeutic effect and identify new efficacy of PMZ. And whether new generation antihistamines have a better ameliorating effect on insulin resistance deserves further investigation.

In summary, our work adds a new perspective to find a novel and shared therapeutic target and drug among ISTs during IR development and will improve our understanding of the development of IR greatly.

## 5 Conclusion

This study revealed Ubd, a novel and shared IR molecular signature across four ISTs, as an effective biomarker and provided new insight into the mechanisms of IR. PMZ was a candidate drug for IR which increased p-Akt level and thus improved IR by targeting Ubd and downregulation of Ubd expression. Both Ubd and PMZ merit further clinical translational investigation to improve IR.

## Data availability statement

The datasets presented in this study can be found in online repositories. The names of the repository/repositories and accession number(s) can be found in the article/ Supplementary material.

## Ethics statement

The animal experimental protocol was approved by the Institutional Animal Care and Use Committee of the School of Medicine of Tongji University (Approved No. TJAA09620206). The study was conducted in accordance with the local legislation and institutional requirements.

## Author contributions

JinX: Data curation, Formal analysis, Methodology, Validation, Visualization, Writing – original draft. LZ: Validation, Writing – original draft. JieX: Resources, Writing – original draft. KL: Methodology, Writing – original draft. JW: Supervision, Writing – review & editing. Y-lB: Supervision, Writing – review & editing. G-TX: Funding acquisition, Supervision, Writing – review & editing. HT: Supervision, Writing – review & editing. FG: Supervision, Writing – review & editing. CJ: Funding acquisition, Supervision, Writing – review & editing. LL: Methodology, Project administration, Supervision, Validation, Writing – review & editing.

## Glossary

**Table:** 

IR	Insulin resistance
Ubd	Ubiquitin D
PMZ	Promethazine
ISTs	Insulin sensitive tissues
GEO	Gene expression omnibus
DEGs	Differentially expressed genes
WGCNA	Weighted gene co-expression network analysis
MSTRGs	Most significant trait-related genes
CTD	Comparative toxicogenomics database
Dex	Dexamethasone
PA	Palmitic acid
dRib	2-deoxy-d-ribose
BAT	Brown adipose tissue
WAT	White adipose tissue
NCD	Normal chow diet
HFD	High fat diet
GO	Gene ontology
BP	Biological process
CC	Cellular component
MF	Molecular function
KEGG	Kyoto encyclopedia of genes and genomes
FA	Fatty acids
PPI	Protein–protein interaction
shRNA	Short hairpin RNA
qRT-PCR	Quantitative reverse transcription polymerase chain reaction
SD	Standard deviation
PTP1B	Protein tyrosine phosphatase-1B

## Footnotes

^1^ http://www.ehbio.com/Cloud_Platform/front/#/analysis?page=b%27MTY%3D%27

^2^ http://diabetes.wisc.edu
