# Supplementary material for: The identification of a novel shared therapeutic target and drug across all insulin-sensitive tissues under insulin resistance
Source: Front Nutr. 2024 Mar 26;11:1381779. doi: 10.3389/fnut.2024.1381779 (PMC11002099; doi:10.3389/fnut.2024.1381779)
Supplement: Supplementary file 1 [file Data_Sheet_1.docx]

***Supplementary Material***

**
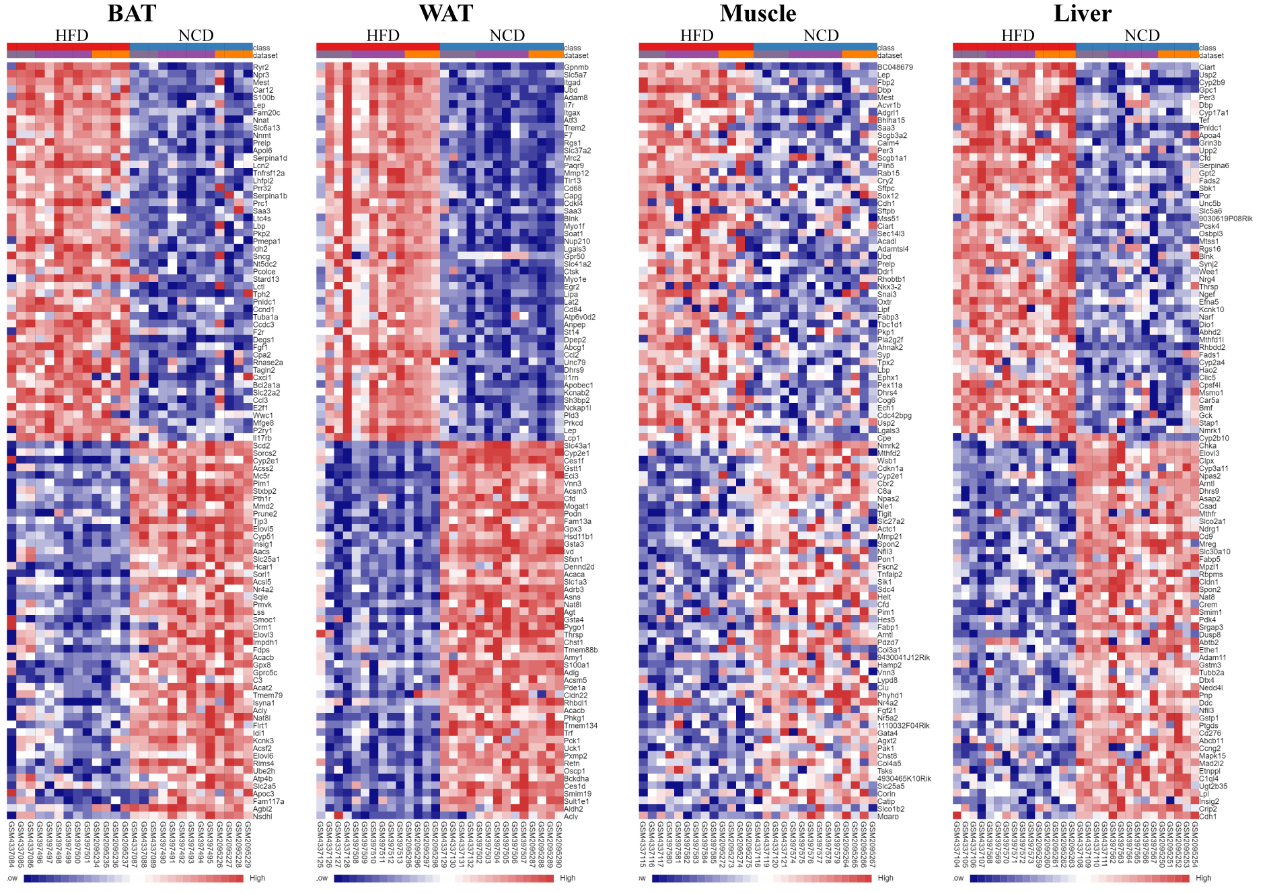
**

**Supplementary Figure 1 Differentially expressed genes analysis.**

Heatmap showing the top 50 upregulated and downregulated DEGs in BAT, WAT, Muscle and Liver in HFD and NCD samples. Red represents upregulated DEGs, blue represents downregulated DEGS, and the gradation of color represents the value of | log FC|**.**

**
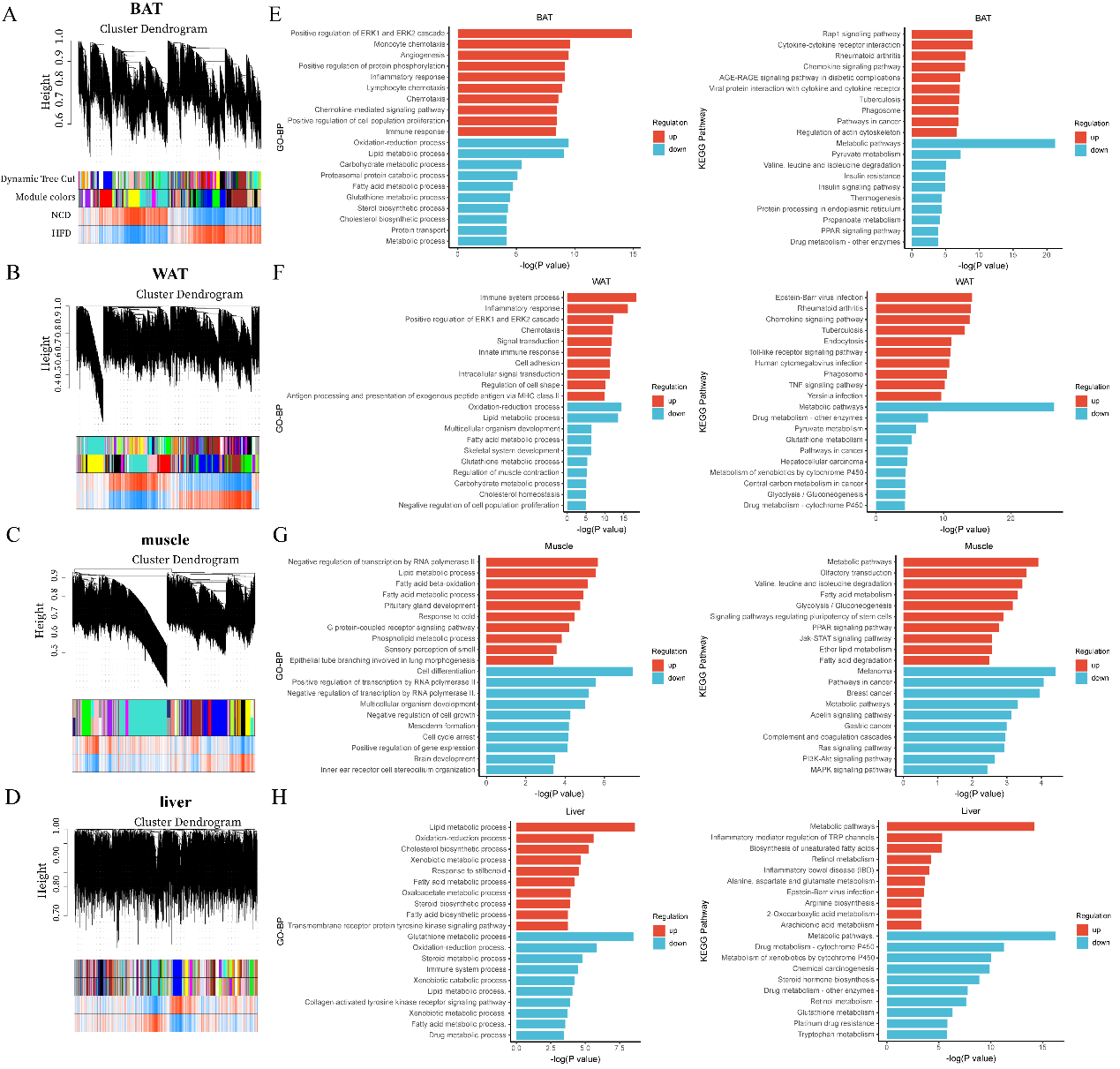
**

**Supplementary Figure 2 The enrichment analysis of MSTRGs in four ISTs.**

(A-D) The cluster dendrogram of the genes with median absolute deviation in the top 5000 of BAT(A), WAT(B), Muscle(C) and Liver (D). Each branch in the figure represents one gene, and every color below represents one co-expression module. (E-H) The top 10 GO-BP terms and KEGG pathways of the MSTRGs (up and down) and the display of top 20 genes of BAT(E), WAT(F), Muscle(G) and Liver (H)**.**


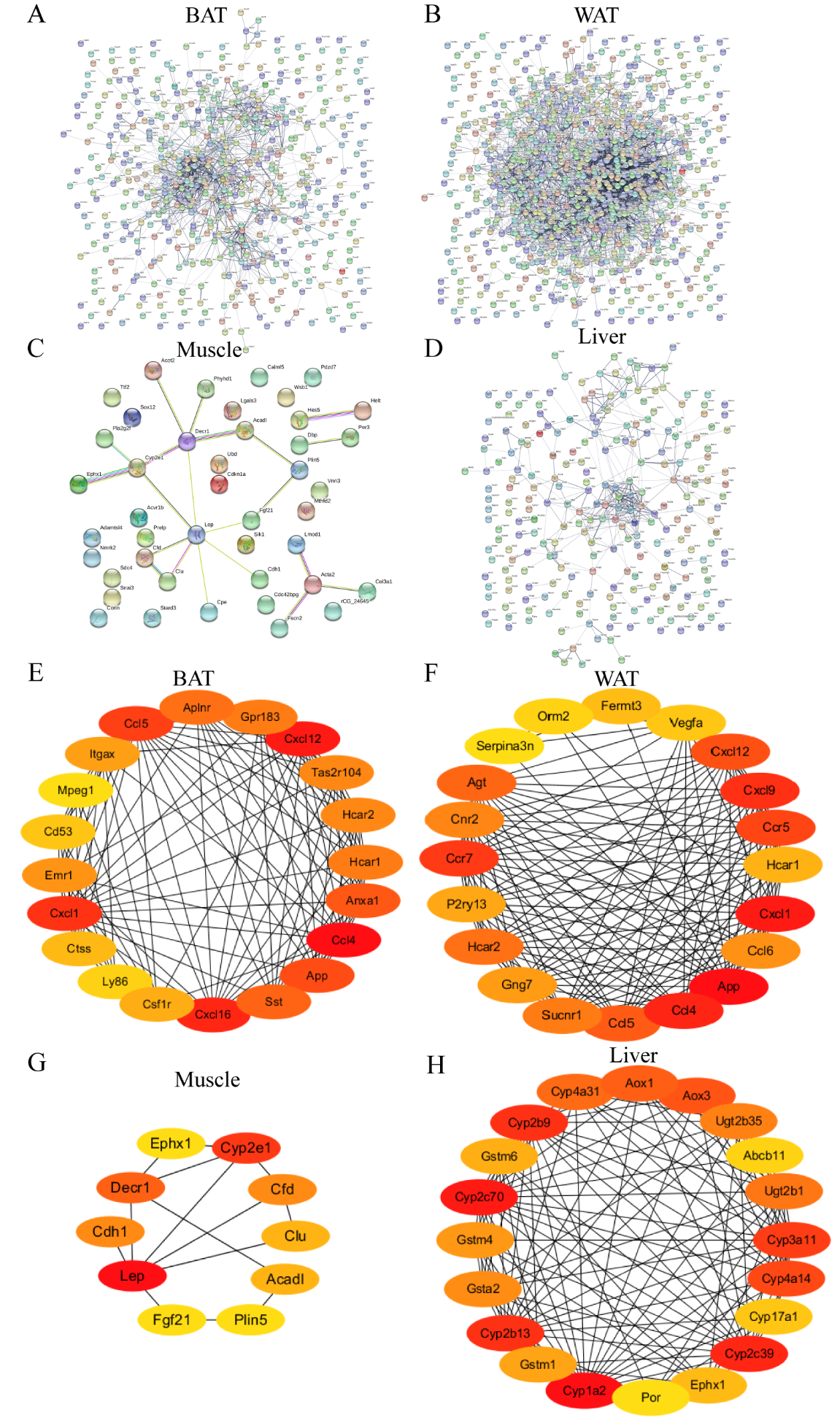


**Supplementary Figure 3 PPI networks and hub genes in ISTs.**

(A-D) The PPI network of shared genes between DEGs and MSTRGs in BAT (A), WAT (B), Muscle (C), and Liver (D). (E-H) The Top 20/10 hub genes of shared genes between DEGs and MSTRGs in BAT (E), WAT (F), Muscle (G), and Liver (H). A node represents a hub target.

**Supplementary Table 1:** **shRNA sequences of mouse ubd**

|  | Forward (5’-3’) | Reverse (5’-3’) |
| --- | --- | --- |
| shubd-1 | GATCCGGCGGTTAATGACCTTTGAGACTTCAAGAGAGTCTCAAAGGTCATTAACCGCTTTTTTG | AATTCAAAAAAGCGGTTAATGACCTTTGAGACTCTCTTGAAGTCTCAAAGGTCATTAACCGCCG |
| shubd-2 | GATCCGCAACATCAAGAGTGGCAGTTTTTCAAGAGAAAACTGCCACTCTTGATGTTGTTTTTTG | AATTCAAAAAACAACATCAAGAGTGGCAGTTTTCTCTTGAAAAACTGCCACTCTTGATGTTGCG |

**Supplementary Table 2:Primers used in RT-PCR experiments**

| Primer | Forward (5’-3’) | Reverse (5’-3’) |
| --- | --- | --- |
| β-actin | ACGGTCAGGTCATCACTATC | GATGCCACAGGATTCCATAC |
| Gpr183 | CAGCTTCTCACGGCAATAA | CCAGACCAATGATGAAGACC |
| Tas2r104 | GTGAAGCTATGCTGGGTATTT | GCCAAGCCAGTGAGAATAAA |
| Gng7 | TGCAGCCACCTTTCATTAG | TATGTCCTTTCCTCTCTCCTC |
| P2ry13 | TCTTCCTGGCAACAACTAAC | GTCTTTGTCCTCCATCTCATAC |
| Cyp2c39 | TGCTTCATTCCTTCCTGAATAG | GAGGGAAGGGACTGGATAAA |
| Aox3 | CCCTGACATCTCACAGAAAC | TGCTGGGAGTCTACATCTT |
| Ugt2b1 | GTGTGGTAGGTGTGGTATTC | GCTCTGCTTCAGCCTTTAT |
| Ugt2b35 | CCTGAGATCACTTGGACATAAC | GCACTTTAGAGCAAGGACTAC |
| Por | GAGCTACGAGAACCAGAAAC | CCAACTCCAGGTGCATTAG |
| Ubd | ACCAGATCCTTCTGCTAGAC | TTAGGGTGAGGTGGATAGTG |
| Lbp | GGGCAGTACGAGTTTCATAG | GAAGAGTCAGAGATGGAGAGA |
| Hp | GAAGTGTGAGCTGCACTATG | AGCACAGAAGGTATGCTTATTC |
| Arntl | GGAAATACGGGTGAAGTCTATG | CCTAGAAGTTCCTGTGGTAGA |
| Cfd | CCTGAACCCTACAAGCATTT | GGAGAGCTTAAAGAGCATGAG |
| Npas2 | GGTACCAAGAGGCTCAATTC | CCTTCTCATCTTCGTCCATTAG |
| Thrsp | CTGGTGTTTCTCTTACCTTCC | CATCCCACACACTCAACTAC |
| Tpx2 | GGCCAGGACTTCAACTATTC | CTAGGACTTGACTGCCTTTATT |
| Pkp1 | CAGTGAGGACATTGAGTGTAG | GGTGTGCTGGATGTAATAGG |
| Sftpd | GAGCCCAACAACAATGGA | GGCTCAGAACTCACAGATAAC |
| Mthfd2 | GGGAATCAACAGTGAGACAA | CAAGGAGGCCATCTACATTC |
| Tnfaip2 | CCCATCTAGGAATAAGCCAAAG | GTACCAACGGGAAGCTAATAAA |
| Vnn3 | GGGACAAGAAGCCATGTAATA | CCTACCCTCAGAATCGAAGA |

**Supplementary Table 3:** **Antibodies used in WB**

| Antibodies | Cat No. | Brand | Dilution ratio |
| --- | --- | --- | --- |
| ubd | A9005 | ABclonal Biotech | 1:1000 |
| p-akt | CY6569 | Abways | 1:1,000 |
| t-akt | CY5551 | Abways | 1:1,000 |
| β-actin | 20536-1-AP | ProteinTech | 1:10,000 |
| GAPDH | 60004-1-Ig | ProteinTech | 1:10,000 |

**Supplementary Table 4. The detail information for known hub genes found in the PPI network in each type of tissues.**

|  | **Rank** | **Name** | **Score** | **Published role in IR** | **Functions in IR** |
| --- | --- | --- | --- | --- | --- |
| BAT | 1 | Ccl4 | 4.79E+08 | PMID: 33968046 | direct inhibition of CCL4 protected pancreatic islet cells, improved IR and retarded the progression of hyperglycemia. |
|  | 2 | Cxcl12 | 4.79E+08 | PMID: 30992469 | CXCL12-CXCR4 pathway activates brown adipocytes and induces IR in CXCR4-deficient mice under high-fat diet. |
|  | 3 | Cxcl16 | 4.79E+08 | PMID: 32535333 | The chemokine CXCL16 can rescue the defects in insulin signaling and sensitivity caused by palmitate in C2C12 myotubes. |
|  | 4 | Cxcl1 | 4.79E+08 | PMID: 29316242 | In plasma, HFD feeding elevated chemokine (C-X-C motif) ligand 1 at day 34 and IL-5 at week 10. |
|  | 5 | Ccl5 | 4.79E+08 | PMID: 23979485 | CCL5 also stimulated insulin secretion from mouse and human islets in vitro, and improved glucose tolerance in lean mice and in a mouse model of hyperglycaemia and IR (ob/ob). |
|  | 6 | App | 4.79E+08 | PMID: 22931791 | IR increases amyloid-β peptide (Aβ) generation. |
|  | 7 | Anxa1 | 4.79E+08 | PMID: 25616869 | annexin A1-formyl peptide receptor 2 (FPR2) pathway in IR. |
|  | 8 | Sst | 4.79E+08 | PMID: 23162532 | Besides their potent regulatory role on GH release, its endocrine actions are highlighted by SST/CORT and ghrelin influence on insulin secretion, glucose homeostasis, and IR. |
|  | 9 | Aplnr | 4.79E+08 | PMID: 29750964 | apelin has certain therapeutic abilities and can be useful in the treatment of, e.g., IR. |
|  | 10 | Gpr183 | 4.79E+08 | N/A | N/A |
|  | 10 | Hcar1 | 4.79E+08 | PMID: 33063454 | DHA reduces serum triglyceride and improves IR and memory exclusively in the fructose-consuming rats. |
|  | 12 | Hcar2 | 4.79E+08 | PMID: 33924461 | Signaling through Hcar2 inhibits adipose tissue lipolysis. |
|  | 12 | Tas2r104 | 4.79E+08 | N/A | N/A |
|  | 14 | Emr1 | 1489458 | PMID: 18270300 | asally, expression of the macrophage markers CD68 and EMR1 were increased in adipose tissue of insulin-resistant subjects. |
|  | 15 | Itgax | 1386755 | PMID: 32928872 | novel loci for many key AT transcripts influencing IR and obesity. |
|  | 16 | Csf1r | 1330999 | PMID: 33564304 | CSF1R, was potential hub genes associated with immune cells' infiltration and the function of proinflammation, especially adipose tissue macrophages, in the progression of obesity-induced diabetes or IR. |
|  | 17 | Ctss | 1228844 | PMID: 35672633 | related to IR, glucose and lipid metabolism |
|  | 18 | Cd53 | 1186568 | PMID: 35672633 | related to IR, glucose and lipid metabolism |
|  | 19 | Ly86 | 1177688 | PMID: 24735745 | DNA methylation of the LY86 gene is associated with obesity, IR, and inflammation. |
|  | 20 | Mpeg1 | 1176846 | PMID： 21687689 | marker genes of macrophage and monocyte (MPEG-1, CD68, F4/80, CD64) were highe |
| WAT | 1 | App | 1.31E+12 | PMID: 22931791 | IR increases amyloid-β peptide (Aβ) generation. |
|  | 2 | Cxcl1 | 1.31E+12 | PMID: 29316242 | In plasma, HFD feeding elevated chemokine (C-X-C motif) ligand 1 at day 34 and IL-5 at week 10. |
|  | 3 | Ccl4 | 1.31E+12 | PMID: 33968046 | direct inhibition of CCL4 protected pancreatic islet cells, improved IR and retarded the progression of hyperglycemia. |
|  | 4 | Cxcl9 | 1.31E+12 | PMID： 31497742 | proinflammatory cytokines |
|  | 5 | Ccr7 | 1.31E+12 | PMID: 26097021 | Protection from diet-induced obesity and IR in mice lacking CCL19-CCR7 signaling. |
|  | 6 | Ccr5 | 1.31E+12 | PMID: 33159935 | Targeting inhibition of CCR5 on improving obesity-associated IR and impairment of pancreatic insulin secretion in high fat-fed rodent models. |
|  | 7 | Cxcl12 | 1.31E+12 | PMID: 30992469 | CXCL12-CXCR4 pathway activates brown adipocytes and induces IR in CXCR4-deficient mice under high-fat diet |
|  | 8 | Ccl5 | 1.31E+12 | PMID: 23979485 | CCL5 also stimulated insulin secretion from mouse and human islets in vitro, and improved glucose tolerance in lean mice and in a mouse model of hyperglycaemia and IR (ob/ob). |
|  | 9 | Agt | 1.31E+12 | PMID: 15699455 | Hypertension genes are genetic markers for insulin sensitivity and resistance. |
|  | 10 | Hcar2 | 1.31E+12 | PMID: 33924461 | Signaling through Hcar2 inhibits adipose tissue lipolysis. |
|  | 11 | Sucnr1 | 1.31E+12 | PMID: 28382382 | SUCNR1-mediated chemotaxis of macrophages aggravates obesity-induced inflammation and diabetes. |
|  | 12 | Cnr2 | 1.31E+12 | PMID: 28895540 | Polymorphism rs3123554 in the cannabinoid receptor gene type 2 (CNR2) reveals effects on body weight and IR in obese subjects. |
|  | 13 | Ccl6 | 1.31E+12 | PMID: 34432833 | involving inflammation. |
|  | 14 | Gng7 | 1.31E+12 | N/A | N/A |
|  | 15 | P2ry13 | 1.31E+12 | N/A | N/A |
|  | 16 | Hcar1 | 1.31E+12 | PMID: 33063454 | DHA reduces serum triglyceride and improves IR and memory exclusively in the fructose-consuming rats. |
|  | 17 | Fermt3 | 9.43E+09 | PMID: 32928872 | novel loci for many key AT transcripts influencing IR and obesity. |
|  | 18 | Vegfa | 7.35E+09 | PMID: 27625023 | enhancing the inflammatory responses, |
|  | 19 | Orm2 | 7.19E+09 | PMID: 33184417 | IR IS mostly encouraged by oxidative stress (CP, TF, ORM2) |
|  | 20 | Serpina3n | 6.71E+09 | PMID: 33654281 | related to several fat expansion and metabolic parameters, including IR index |
| Muscle | 1 | Lep | 9 | PMID: 22883229 | Selective insulin and leptin resistance in metabolic disorders. |
|  | 2 | Cyp2e1 | 8 | PMID: 35662646 | IR enhances binge ethanol-induced liver injury through promoting oxidative stress and up-regulation CYP2E1. |
|  | 3 | Decr1 | 6 | PMID： 23244828 | affect lipid metabolism |
|  | 4 | Cfd | 4 | PMID: 31700183 | Adipsin preserves beta cells in diabetic mice and associates with protection from type 2 diabetes in humans. |
|  | 4 | Cdh1 | 4 | PMID: 32378562 | DOE increased E-cadherin，accelerating lipid transport, inhibiting insulin resistant and suppressing fibrosis induced by epithelial mesenchymal transition (EMT). |
|  | 6 | Clu | 3 | PMID: 30659075 | Clusterin Impairs Hepatic Insulin Sensitivity and Adipocyte Clusterin Associates With Cardiometabolic Risk. |
|  | 6 | Acadl | 3 | PMID: 34638602 | ACADL) were the direct target genes of miR-126b-5p, MicroRNA-126b-5p Exacerbates Development of Adipose Tissue and Diet-Induced Obesity. |
|  | 8 | Ephx1 | 2 | PMID: 34342583 | CRISPR-Cas9-mediated EPHX1 knockout (KO) abolished adipocyte differentiation and decreased insulin response |
|  | 8 | Fgf21 | 2 | PMID: 29987000 | FGF21 gene therapy as treatment for obesity and IR. |
|  | 8 | Plin5 | 2 | PMID: 25161888 | PLIN5 deletion remodels intracellular lipid composition and causes IR in muscle. |
| Liver | 1 | Cyp1a2 | 7326250 | PMID: 33479273 | High IR states could be associated with CYP1A2 induction |
|  | 2 | Cyp2c70 | 7325610 | PMID: 27638959 | oxidation of CDCA and UDCA by Cyp2c70 |
|  | 3 | Cyp2c39 | 7325280 | N/A | N/A |
|  | 4 | Cyp2b9 | 7318416 | PMID: 12130701 | mRNA levels of Cyp2b9 was increased markedly in liver from diabetic mice with no or only a slight increase in insulin resistant mice. |
|  | 4 | Cyp2b13 | 7318416 | N/A | N/A |
|  | 6 | Cyp3a11 | 7280670 | PMID: 26219821 | higher Cyp3a11 |
|  | 7 | Cyp4a14 | 7263200 | PMID: 30088983 | High-fat diet-induced obesity and IR in CYP4a14-/- mice is mediated by 20-HETE. |
|  | 8 | Aox3 | 7257728 | N/A | N/A |
|  | 9 | Aox1 | 7257722 | PMID: 34981123 | associated with lipid synthesis, fatty acid oxidation, and hepatocyte steatosis |
|  | 10 | Cyp4a31 | 7257720 | N/A | N/A |
|  | 11 | Ugt2b1 | 3635059 | N/A | N/A |
|  | 12 | Ugt2b35 | 3634722 | N/A | N/A |
|  | 13 | Gsta2 | 62952 | PMID: 24007921 | hepatic expression of FXR and xenobiotic gene |
|  | 14 | Gstm4 | 51384 | PMID: 20119733 | NASH patients showed over-expression of GSTM 2, GSTM4 and GSTM5 as well as FH and ASCL4 genes |
|  | 15 | Gstm1 | 45602 | PMID: 22732554 | GSTM1, GSTT1, and GSTP1 polymorphisms and associations between air pollutants and markers of insulin resistance in elderly Koreans. |
|  | 16 | Gstm6 | 45480 | PMID: 12130701 | expression of Cyp1a2, Cyp7b1, Gstm3, and Gstm6 was reduced in both diabetic and insulin resistant mice |
|  | 17 | Ephx1 | 7536 | PMID: 34342583 | CRISPR-Cas9-mediated EPHX1 knockout (KO) abolished adipocyte differentiation and decreased insulin response |
|  | 18 | Cyp17a1 | 5226 | PMID: 32510471 | selectively targeting hepatic Cyp17A1 may provide a therapeutic avenue for treating T2DM. |
|  | 19 | Abcb11 | 396 | PMID： 21386085 | ssociated with HDLC-GLUC |
|  | 20 | Por | 265 | N/A | N/A |

**Supplementary Table 5. The detail information for betweenness centrality genes found in the PPI network in each type of tissues.**

|  | **Rank** | **Name** | **Betweenness centrality** | **Published role in IR** | **Functions in IR** |
| --- | --- | --- | --- | --- | --- |
| BAT | 1 | Cdk1 | 2.89E+04 | PMID: 33345777 | a diabetic-like phenotype caused by loss of CDK1 |
|  | 2 | Decr1 | 2.63E+04 | PMID: 33297922 | DECR1 the liver-brain axis |
|  | 3 | Ccnd1 | 2.53E+04 | PMID: 25073444 | HP-fed mice were obese and insulin resistant, which had increased levels of Ccnd1 mRNA |
|  | 4 | App | 1.58E+04 | PMID: 29058763 | abnormal insulin signaling is connected with cognitive dysfunction in AD model using APP/PS1 transgenic mice |
|  | 5 | Lep | 1.51E+04 | PMID: 36228616 | Lep is associated with obesity and insulin resistance |
|  | 6 | Grb2 | 9.82E+03 | PMID: 36176460 | GRB2 concentrations were positively associated with duration of diabetes, FINS, homeostasis model assessment-insulin resistance index (HOMA-IR) |
|  | 7 | Ccl2 | 9.51E+03 | PMID: 26839895 | CCL2 Serum Levels and Adiposity Are Associated with the Polymorphic Phenotypes -2518A on CCL2 and 64ILE on CCR2 in a Mexican Population with Insulin Resistance |
|  | 8 | Ctla4 | 8.53E+03 | PMID: 23872146 | CTLA-4Ig immunotherapy of obesity-induced insulin resistance by manipulation of macrophage polarization in adipose tissues |
|  | 9 | Cdh5 | 7.88E+03 | PMID: 19997558 | the association of CDH5 with β-catenin might play a significant role in diabetes mellitus through the impairment of vascular endothelial cell function |
|  | 10 | Bmp4 | 7.87E+03 | PMID: 32029225 | BMP4 gene therapy enhances insulin sensitivity but not adipose tissue browning in obese mice |
| WAT | 1 | Cd44 | 3.12E+04 | PMID: 35249813 | recent evidence suggests a role of CD44 in metabolism, especially insulin resistance in obesity and diabetes |
|  | 2 | Apob | 2.71E+04 | PMID: 23721961 | Insulin plays a key role in the regulation of ApoB. Insulin decreases ApoB secretion by promoting ApoB degradation in the hepatocyte |
|  | 3 | Vegfa | 2.67E+04 | PMID: 29196658 | Vascular endothelial growth factor A (VEGFA) is a key factor in the regulation of angiogenesis in adipose tissue |
|  | 4 | Mmp9 | 2.57E+04 | PMID: 24305966 | genetic deletion of MMP9 in mice increases muscle ColIV, induces insulin resistance in lean mice and worsens diet-induced muscle insulin resistance |
|  | 5 | Rem1 | 2.45E+04 | N/A | N/A |
|  | 6 | App | 2.25E+04 | PMID: 29058763 | abnormal insulin signaling is connected with cognitive dysfunction in AD model using APP/PS1 transgenic mice |
|  | 7 | Ccnb1 | 2.24E+04 | PMID: 21330319 | The decrease in IGF-IR by siRNA was associated with decreases in pAkt and treatment of MCL cell lines with IGF-IR siRNA increased cyclin B1 |
|  | 8 | Rab7 | 2.05E+04 | PMID: 36959859 | Celastrol directly binds with VAMP7 and RAB7 to inhibit autophagy and induce apoptosis in preadipocytes |
|  | 9 | H6pd | 1.81E+04 | PMID: 22306327 | Both H6PD variants were associated with several phenotypic variables, including fasting insulin, homeostasis model assessment of insulin resistance |
|  | 10 | Tlr2 | 1.66E+04 | PMID: 36555322 | TLR2 knock-out mice spontaneously developed mature-onset obesity and insulin resistance |
| Muscle | 1 | Lep | 193.66667 | PMID: 36228616 | Lep is associated with obesity and insulin resistance |
|  | 2 | Decr1 | 99.333336 | PMID: 33297922 | DECR1 the liver-brain axis |
|  | 3 | Cdh1 | 96 | PMID: 29941485 | E-cadherin (CDH1) as a repressor of IGF1 signaling |
|  | 4 | Cyp2e1 | 81.666664 | PMID: 35662646 | Insulin resistance enhances binge ethanol-induced liver injury through promoting oxidative stress and up-regulation CYP2E1 |
|  | 5 | Acadl | 43 | PMID: 31577934 | Dietary Sugars Alter Hepatic Fatty Acid Oxidation via Transcriptional and Post-translational Modifications of Mitochondrial Proteins |
|  | 6 | Clu | 34 | PMID: 37415417 | Clusterin is closely associated with adipose tissue insulin resistance |
|  | 7 | Fgf21 | 21 | PMID: 29987000 | FGF21 gene therapy as treatment for obesity and insulin resistance |
|  | 8 | Cfd | 7.3333335 | PMID: 30153273 | CFD induced obesity after a 6-week diet treatment, glucose intolerance and insulin resistance after a 16-week-diet treatment |
|  | 9 | Plin5 | 4 | PMID: 25161888 | Share   PLIN5 deletion remodels intracellular lipid composition and causes insulin resistance in muscle |
|  | 10 | Actc1 | 2 | N/A | N/A |
| Liver | 1 | Egfr | 7258.273 | PMID: 35948530 | EGFR-mediated activation of adipose tissue macrophages promotes obesity and insulin resistance |
|  | 2 | Apoa4 | 3832.226 | PMID: 35909347 | Apolipoprotein A4 Restricts Diet-Induced Hepatic Steatosis via SREBF1-Mediated Lipogenesis and Enhances IRS-PI3K-Akt Signaling |
|  | 3 | Pcsk9 | 3198.075 | PMID: 28587771 | PCSK9 appears to modulate glucose intolerance, insulin resistance, abdominal obesity, inflammation, and hypertension |
|  | 4 | Hspa5 | 3073.6353 | PMID: 36513656 | Upper gut heat shock proteins HSP70 and GRP78 promote insulin resistance, hyperglycemia, and non-alcoholic steatohepatitis |
|  | 5 | C8b | 2787.8657 | PMID: 7665162 | two SSRs, (GA)n and (GT)n, near the rat complement component 8β gene (C8b) at the rat fatty locus (fa, facp), which produce obesity, insulin resistance, and diabetes, provide useful experimental models for similar phenotypes in humans |
|  | 6 | Nr1d1 | 2661.7234 | PMID: 34350828 | NR1D1 action in adipocytes is critical to the development of obesity-related WAT pathology and insulin resistance |
|  | 7 | Ugt2b1 | 2319.8408 | N/A | N/A |
|  | 8 | G6pc | 1921.9287 | PMID: 25685698 | Intestinal-G6pc (-/-) mice exhibit slight fasting hyperglycaemia and hyperinsulinemia, glucose intolerance, insulin resistance |
|  | 9 | Cyp2c70 | 1859.5664 | PMID: 27638959 | Cyp2c70 is responsible for the species difference in bile acid metabolism between mice and humans |
|  | 10 | Gamt | 1642.9697 | PMID: 36791500 | Gamt is down-regulated in LKO mice |
